# Role of Reversed Austenite Behavior in Determining Microstructure and Toughness of Advanced Medium Mn Steel by Welding Thermal Cycle

**DOI:** 10.3390/ma11112127

**Published:** 2018-10-29

**Authors:** Yunxia Chen, Honghong Wang, Huan Cai, Junhui Li, Yongqing Chen

**Affiliations:** 1 School of Materials Science and Engineering, Shanghai Dianji University, Shanghai 201306, China; chenyx@sdju.edu.cn; 2 The State Key Laboratory of Refractories and Metallurgy, Wuhan University of Science and Technology, Wuhan 430081, China; caihuan7963@163.com (H.C.); 15671628834@163.com (J.L.); chenyqwork@163.com (Y.C.)

**Keywords:** reverse transformation, grain growth of reversed austenite, welding thermal cycle, advanced medium Mn steels, impact toughness

## Abstract

Reversed austenite transformation behavior plays a significant role in determining the microstructure and mechanical properties of heat affected zones of steels, involving the nucleation and growth of reversed austenite. Confocal Laser Scanning Microscope (CLSM) was used in the present work to in situ observe the reversed austenite transformation by simulating welding thermal cycles for advance 5Mn steels. No thermal inertia was found on cooling process after temperature reached the peak temperature of 1320 °C. Therefore, too large grain was not generated in coarse-grained heat-affected zone (CGHAZ). The pre-existing film retained austenite in base metal and acted as additional favorable nucleation sites for reversed austenite during the thermal cycle. A much great nucleation number led to the finer grain in the fine-grained heat-affected zone (FGHAZ). The continuous cooling transformation for CGHAZ and FGHAZ revealed that the martensite was the main transformed product. Martensite transformation temperature (Tm) was higher in FGHAZ than in CGHAZ. Martensite transformation rate was higher in FGHAZ than in CGHAZ, which is due to the different grain size and assumed atom (Mn and C) segregation. Consequently, the softer martensite was measured in CGHAZ than in FGHAZ. Although 10~11% austenite retained in FGHAZ, the possible Transformation Induced Plasticity (TRIP) effect at −60 °C test temperature may lower the impact toughness to some degree. Therefore, the mean absorbed energy of 31, 39 and 42 J in CGHAZ and 56, 45 and 36 J in FGHAZ were exhibited at the same welding heat input. The more stable retained austenite was speculated to improve impact toughness in heat-affected zone (HAZ). For these 5Mn steels, reversed austenite plays a significant role in affecting impact toughness of heat-affected zones more than grain size.

## 1. Introduction

Grain size plays a significant role in determining the strength and toughness of materials. During the welding process, austenite tends to grow thermally and the austenite grain size provides the initial condition for the subsequent phase transformation during cooling and then affects the final microstructure and resulting mechanical properties [1,2]. It is reported that increasing the austenite grain size shifts the continuous cooling transformation diagram to longer reaction time and increases the possibility of martensite formation. Martensite formation may lower the toughness [3,4,5]. Large austenite grain size is of particular concern during welding where the HAZ experiences rapid thermal cycles with high peak temperature which give rise to austenite grain growth, especially in the region adjacent to the fusion zone (coarse-grained heat-affected zone, CGHAZ). Therefore, the austenite growth behavior has aroused much interest in the past several decades [6,7,8].

Alloying elements are of importance in affecting the austenite grain growth. Microalloying elements, like Nb, V and Ti, suppress the austenite growth as (Nb,V,Ti)(C,N) precipitates pin the austenite grain boundaries before complete dissolution at 1200 °C on heating [9,10,11,12]. Some elements, like B, easily segregate at grain boundaries. Thus, they reduce the austenite growth rate and result in finer grain at room temperature in CGHAZ. Ni is an austenite former element. When enriched in retained austenite in the original microstructure, it may enhance the reversed austenite nucleation site and lead to the final finer grain [13,14]. Other main elements, e.g., Mn should play a role in influencing the austenite grain growth due to either austenite former element or segregation to the grain boundaries. However, there is a lack of information about the Mn effect on austenite growth during welding.

Mn has been utilized in advanced cryogenic steels to replace Ni recently. Like Ni, Mn can enlarge the γ phase and prompt reversed austenite. Additionally, Mn enriches the reversed austenite and enhances its stability, thus improving the cryogenic toughness. In this present work, 5% Mn cryogenic steel was experimented. The following issues were investigated based on reversed austenite transformation at a high temperature of the welding thermal cycle:

(1) The reversed austenite nucleation characterization on heating.
(2) The reversed austenite growth kinetic.
(3) The effect of different austenite grain size on continuous cooling transformation temperature, and thus martensite transformation and reversed austenite transformation.
(4) The role of microstructure in influencing the cryogenic toughness of heat-affected zone.

## 2. Experimental

The material studied is an advanced medium Mn steel. Its chemical composition is shown in Table 1 and its mechanical properties are shown in Table 2. The microstructure consists of main martensite and 10~15 vol.% retained austenite. The high yield strength of 650 MPa, ultimate strength of 770 MPa, and superior cryogenic toughness of around 200 J at −60 °C were obtained by optimized intercitical annealing. The transformation temperature was measured as Ac1 = 626 °C and Ac3 = 790 °C on a slow heating rate of 3 °C/min by Gleeble 3800 (Dynamic Systems Inc., Poestenkill, NY, USA) [15].

A He–Ne CLSM (VL2000DX-SVF17SP, Lasertech Yokohama, Chiba, Japan) with infrared image furnace and laser scanning confocal microscopy was employed to in situ observe the reverse transformation of austenite on heating, and martensite transformation on cooling during the simulated welding thermal cycle. The CLSM samples, with approximate 4 mm in diameter and 6 mm in height, were carefully machine polished, and then set into an alumina crucible with 0.5 mm in thickness. The sample chamber was evacuated and then filled with argon to prevent the sample from being oxidized during heating. Using focused infrared light heating mode, the specimens were heated to the peak temperature of 1320 °C at the rate of 5 °C·s^−1^, and then cooled down to 200 °C at the rate of 5 °C·s^−1^. The live pictures were taken every 15 s from 240 mm × 240 mm surface areas.

Gleeble 3800 was used to detect the expansion variation during the simulated welding thermal cycle. The sample was 6 mm in diameter and 70 mm in length. The peak temperature was 1320 and 850 °C to simulate the coarse-grained heat affected zone (CGHAZ) and fine-grained heat affected zone (FGHAZ) respectively. The different cooling rates of 1~60 °C·s^−1^ were set to draw the simulated heat-affected zone continuous cooling transformation diagram (SHCCT). The transformation temperature and transformation kinetic were detected based on the thermal expansion curves.

The mean grain size was estimated on the CLSM sample using a circular-intercept method from image analysis of at least 500 grains. Intercept lengths were determined and then converted into nominal grain diameters using standard tables. The electron backscattering diffraction (EBSD) was applied to analyze the misorientation of the microstructure.

The Metallographic specimens of simulated HAZs were polished and etched with 2% nital before conventional light microscopy. Transmission electron microscopy (TEM, JEM-2100UHR STEM/EDS, JEOL Ltd., Tokyo, Japan) studies were carried out on thin foils. Thin foils were prepared by cutting thin wafers from the simulated HAZ samples, and grinding to 0.1 mm in thickness. Three millimeter discs were punched from the wafers, and were electropolished using a solution of 5% perchloric acid/95% acetic acid. Foils were examined by conventional transmission electron microscope operated at 120 kV using standard bright field and dark field imaging techniques.

The volume fraction of reversed austenite was determined using XRD (XPert PRO MPD, PANalytical B. V., Almelo, Holland) with Cu Kα radiation at a scanning speed of 1°·min^−1^ and step size of 0.02°. The specimens of simulated HAZ were first mechanically polished and then electropolished using an electrolyte consisting of 12% perchloric acid and 88% absolute ethyl alcohol at room temperature. The integrated intensities of (200)γ, (220)γ, (311)γ, (110)α, (200)α, (211)α, (220)α diffraction peaks were used to determine the austenite volume fraction.

Hardness was measured on simulated HAZ samples with a load of 200 g, using a MICROMET5101 Vickers hardness tester (Matsuzawa Co., Ltd., Tokyo, Japan). Standard Charpy v-notch (CVN) impact tests were performed on simulated samples with a different cooling rate at −60 °C, using a NI500A imapct tester (NCS Co., Ltd., Beijing, China) on specimens of dimensions 10 × 10 × 55 mm^3^.

## 3. Results and Discussion

### 3.1. Nucleation and Growth of Reversed Austenite

**Figure 1:**
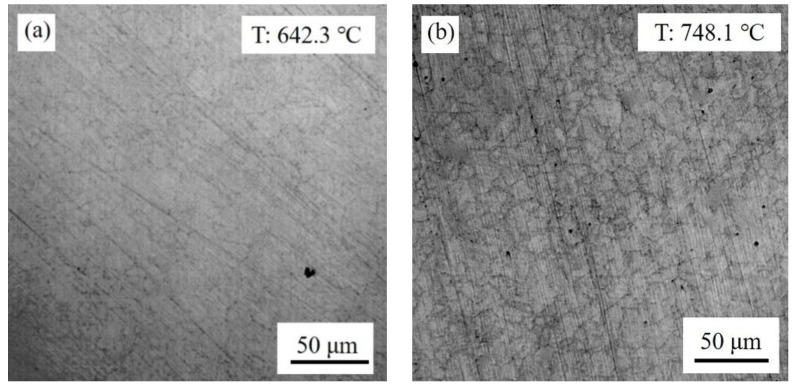

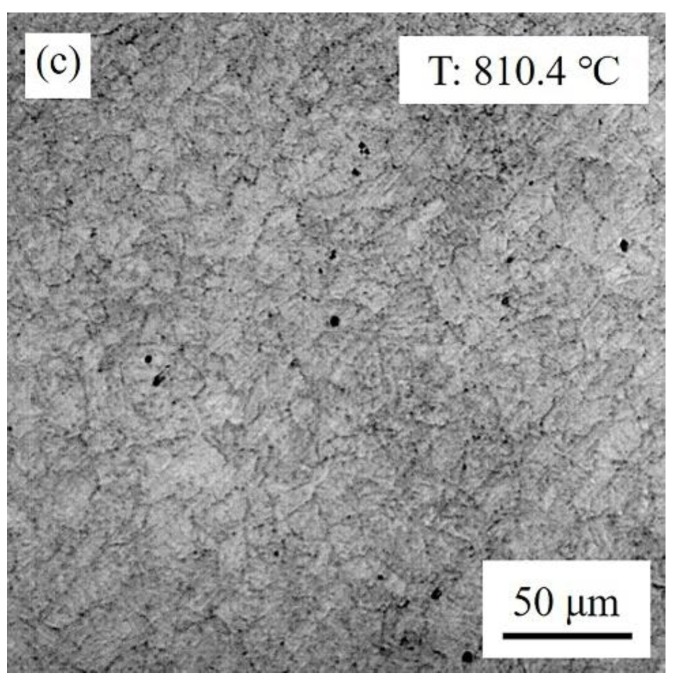
Reversed austenite at different temperatures on heating during thermal cycle: (**a**) 642.3 °C, (**b**) 748.1 °C, (**c**) 810.4 °C.

Figure 1a shows that reversed austenite started to nucleate when the temperature reached around 640 °C. Due to the limitation of magnification, only nucleation at the grain boundaries was observed clearly; the nucleation inside the grain cannot be presented. Theoretically, the pre-existing retained austenite were the preferable sites for austenite nucleation and growth [16]. In this investigated steel, the 5~10% retained austenite played a beneficial role in improving nucleation number. Therefore, after completion of reversed austenite transformation, the grain size was small. As seen in Figure 1b, the grain size was around 3~10 μm at the temperature of 850 °C.

**Figure 2:**
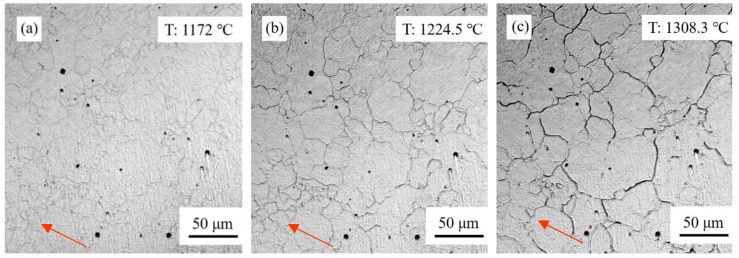
Austenite growth at different temperatures on heating during thermal cycle: (**a**) 1172 °C, (**b**) 1224.5 °C, (**c**) 1308.3 °C.

**Figure 3:**
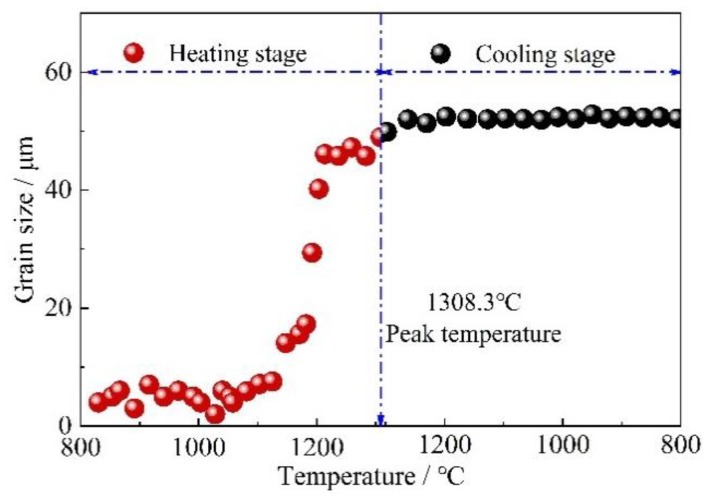
Austenite grain size VS temperature in the heating and cooling stage.

After completion of reversed austenite transformation on heating, austenite started to grow, as shown in Figure 2. The austenite grain size at different temperatures was measured on the CLSM samples using the circular-intercept method. The austenite growth rate was plotted in Figure 3. It reveals that the grain size growth rate was small below the temperature of 1100 °C on continuous heating. There was a sharp increase in austenite growth from 1100 °C up to 1250 °C. When the peak temperature of 1308 °C was attained, the maximum grain size of ~52 μm was observed and almost no growth was found during the following continuous cooling until it reached austenite decomposition temperature.

The reversed austenite generally nucleated at grain boundaries, as seen in Figure 1. Especially, the pre-existing retained austenite took a positive role in improving austenite nucleation sites. The filmy retained austenite grew directly in one dimension, which implies a larger nucleation number in the present investigated steel. The large initial nucleation number caused the slow grain growth. On the other hand, the grain grew in the boundary migration way, which is controlled by the relatively short distance of carbon diffusion. Therefore, the grain growth rate was small below 1100 °C.

During the temperature increases, the element diffusion coefficient became lager. The large diffusion of both carbon and Mn changed the method of grain growth from grain boundary migration to grain annexation. The sharp increase of grain growth rate was present during continuous heating from 1100 °C up to 1250 °C.

In low-alloyed steel, the austenite grain continuously grows up during following cooling process above peak temperature [2], which is referred to as “hot-inertia” [17]. Interestingly, in this present steel, there was almost no growth of austenite measured, as shown in Figure 3. It is reported that Mn atoms are likely to segregate toward grain boundaries during continuous cooling [18]. Mn segregation at the grain boundary was assumed to pin the grain boundary migration and hinder the growth of austenite grain. Further study is needed to figure out the Mn segregation behavior and its effect on grain growth.

### 3.2. Effect of Prior Austenite Grain Size on the Continuous Cooling Transformation Temperature and Transformation Rate

**Figure 4:**
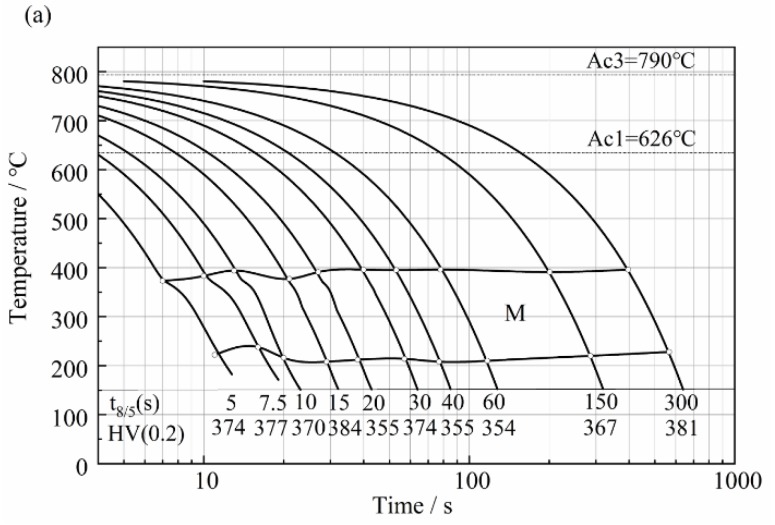

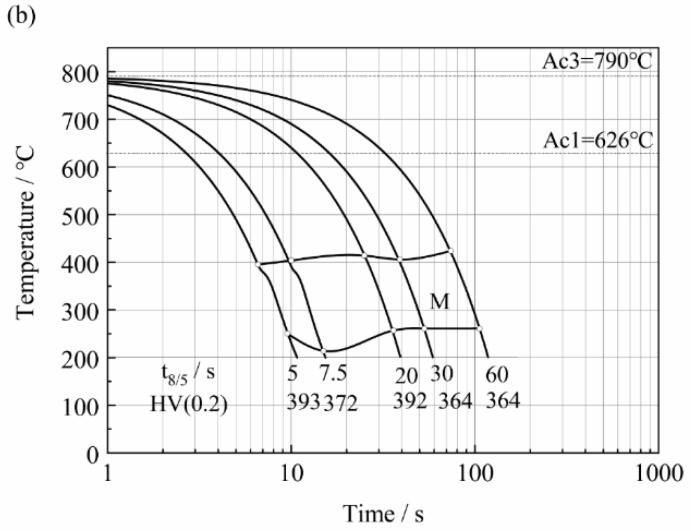
Simulated heat-affected zone continuous cooling transformation diagram for peak temperature of (**a**) 1320 °C; (**b**) 850 °C.

Figure 4 shows the simulated heat-affected zone continuous cooling transformation diagram. Figure 4a is a SHCCT diagram for coarse-grained HAZ with the peak temperature of 1320 °C, while Figure 4b is for fine-grained HAZ with the peak temperature of 850 °C. Both diagrams indicate that martensite was the main phase. However, the martensite’s start transformation temperature in FGHAZ was higher than that in CGHAZ by 17~31 °C at the same cooling rate, while the finish transformation temperature in FGHAZ was higher than that in CGHAZ by 31~58 °C, with an exception at the t_8/5_ of 7.5 s. Moreover, the transformation temperature range in FGHAZ was smaller than that in CGHAZ, as shown in Table 3.

Mn has a definite segregation tendency due to its differential diameter of 5 × 10^−12^ m with the Fe element. During the welding thermal cycle, Mn may segregate towards gain boundaries by the nonequilibrium segregation mechanism. The higher the peak temperature is, the higher the segregation concentration of Mn at grain boundaries is. For FGHAZ, the small grain size allows Mn and C to diffuse short distance to grain boundary. Furthermore, the great number of grain boundaries can enhance more Mn and C segregates at the grain boundaries. Therefore, the concentration of Mn and C should be lower in FGHAZ than in CGHAZ. Consequentially, the martensite start temperature was higher in FGHAZ than in CGHAZ.

**Figure 5:**
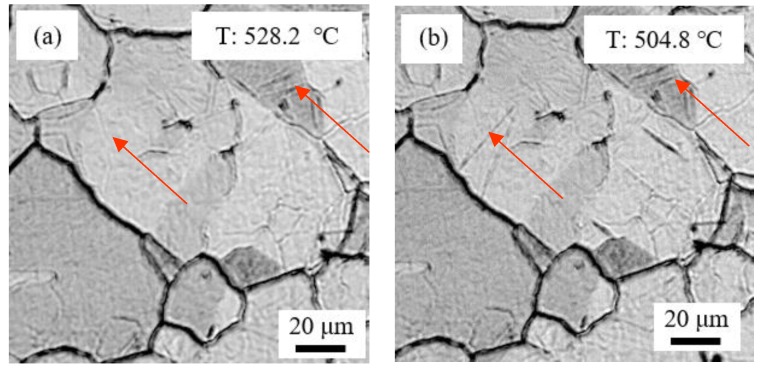

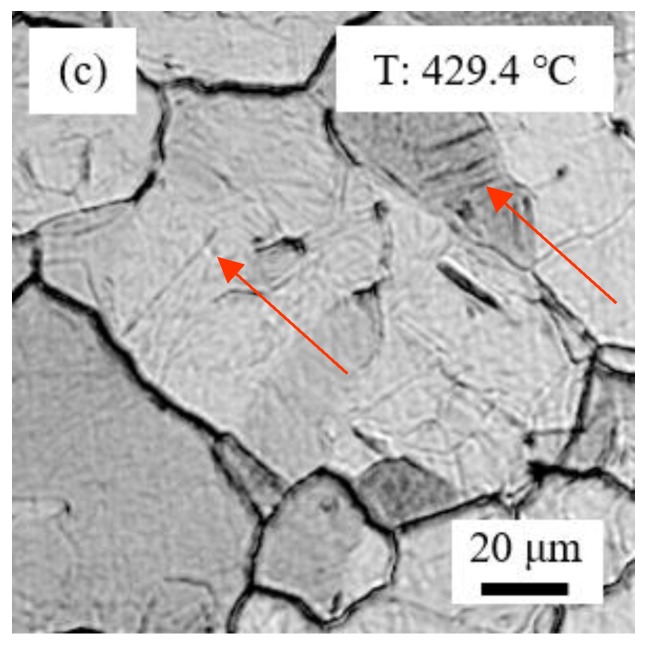
Martensite transformation in the cooling stage in CGHAZ at different temperature of (**a**) 528.2 °C, (**b**) 504.8 °C, (**c**) 429.4 °C.

The in situ observation of martensite transformation by SLCM (Figure 5) shows that lath martensite nucleated inside austenite grain and then grew quickly along the length direction. The austenite was divided by the first generated longer lathes. Then the lath widened. In the fine austenite grain, the martensite grew in the limited space, so short and thin martensite lath was found in FGHAZ. On the other hand, long and thick martensite lath was found in CGHAZ.

**Figure 6:**
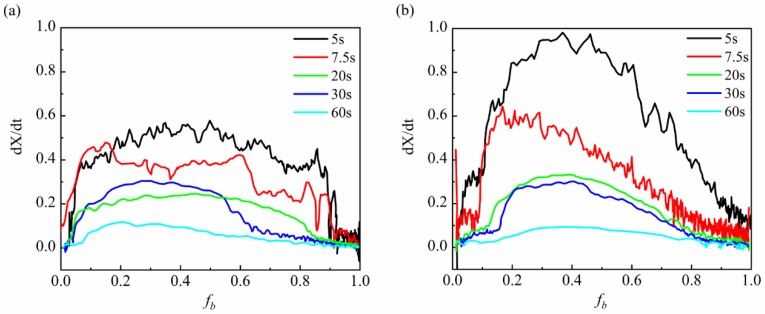
Martensite transformation rate VS martensite transformed volume fraction with different cooling rate at peak temperatures of: (**a**) 1320 °C, (**b**) 850 °C.

Figure 6 reveals the martensite transformation rate vs martensite transformed volume fraction. At the peak temperature of 1320 °C, the transformation rate from austenite to martensite was measured to be a maximum of 0.60, while at the peak temperature of 850 °C, the transformation rate from austenite to martensite was measured to be a maximum of 0.98. The transformation rate is closely related to the transformation start temperature and transformation temperature range. The higher start transformation temperature often leads to a relatively high transformation rate. Therefore, because the martensite transformation temperature was higher at the peak temperature of 850 °C than that at 1320 °C, as well as the grain size was smaller at the peak temperature of 850 °C than that at 1320 °C, FGHAZ expressed the relatively higher transformation rate.

### 3.3. Martensite Transformation and Retained Austenite

**Figure 7:**
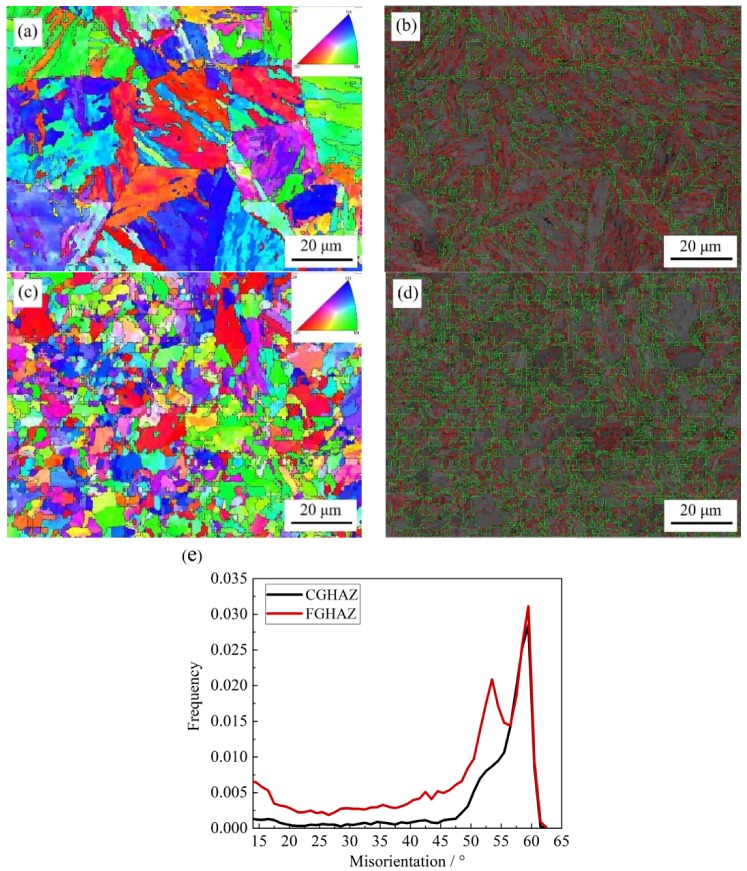
Crystallographic analysis in sample Z direction of simulated (**a**) CGHAZ, (**c**) FGHAZ, EBSD superimposed figure of band contrast and grain boundaries (misorientation < 15° in red line, misorientation > 15° in green line) of simulated (**b**) CGHAZ, (**d**) FGHAZ with t_8/5_ of 25 s. (**e**) Curves of misorientation angle-relative frequency of simulated HAZs.

Figure 7 shows the crystallography of CGHAZ and FGHAZ by EBSD. It is clear that CGHAZ had the larger grain (Figure 7a) compared with FGHAZ (Figure 7c). The EBSD superimposed figure in Figure 7b–d of band contrast and grain boundaries indicated that the number of boundaries with the misorientation >15° in FGHAZ was greater than in CGHAZ, which means that the effective grain size in FGHAZ was smaller than that in CGHAZ. The comparison is presented in Figure 7e. This agreed well with the in situ observation of martensite transformation.

**Figure 8:**
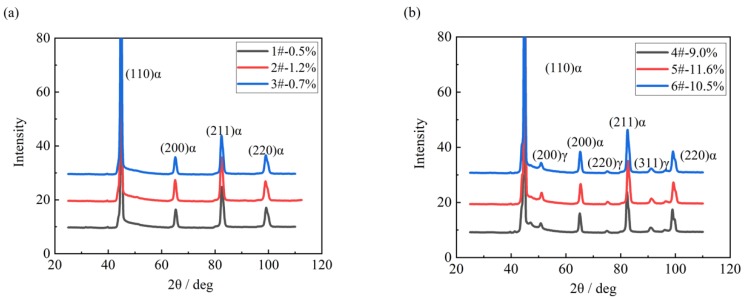
Comparison of volume fraction of retained austenite by XRD in simulated (**a**) CGHAZ and (**b**) FGHAZ.

Apart from martensite transformation, some austenite was retained in FGHAZ because of the fast continuous cooling transformation (shown in Figure 4), as well as the shorter transformation temperature range. The volume fraction of retained austenite in FGHAZ was measured to be 9~11% by XRD, while only 0.5~1.2% retained austenite in CGHAZ (Figure 8a,b).

**Figure 9:**
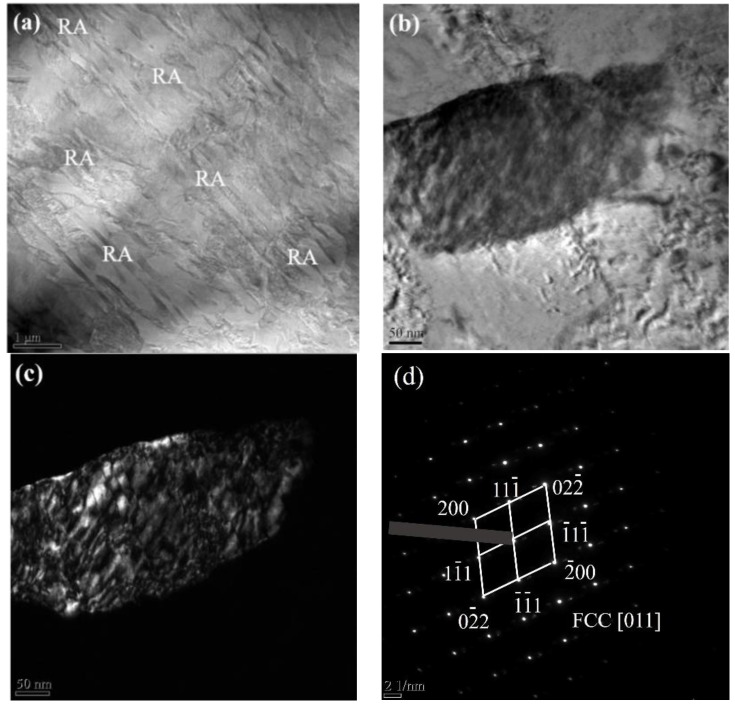
TEM showing (**a**) RA morphology, (**b**) Bright field micrograph and (**c**) Dark field micrograph (**d**) diffraction pattern showing austenite in simulated FGHAZ at t_8/5_ of 10 s.

**Figure 10:**
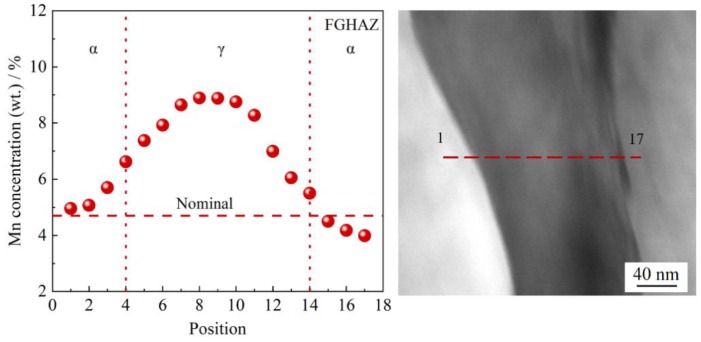
TEM micrograph of retained austenite and EDS line-scan along the red line in retained austenite of simulated FGHAZ at t_8/5_ of 10 s.

The retained austenite morphology in FGHAZ by TEM is presented in Figure 9. The retained austenite was found along the martensite lath, as shown in Figure 9a. In the bright field micrograph (Figure 9b), the retained austenite was found to be film-like, and thickness was around ~150 nm. The retained austenite is demonstrated by the field micrograph in Figure 9c and the diffraction pattern in Figure 9d. By using EDS line-scan, the concentration of Mn in retained austenite was measured and there was ~1.8 enrichment factor of Mn found in retained austenite, which is shown in Figure 10.

The enrichment of Mn of 9% in retained austenite lowers the martensite transformation temperature. It is possible for retained austenite to transform into martensite in an impact toughness test temperature of −60 °C due to the lower stability of retained austenite.

### 3.4. Hardness

The SHCCT diagram in Figure 4 shows that the hardness of FGHAZ was around 363~393 HV (0.2) and around 354~384 HV (0.2) of CGHAZ. Both at the peak temperature of 1320 °C and 850 °C, the continuous cooling transformed product was martensite. Figure 4 also reveals that at the same t_8/5_ range of 5~60 s, each average hardness value of FGHAZ was larger than that of CGHAZ by around 10 HV. The harder martensite in FGHAZ was attributed to the higher transformation rate, as well as fine grain.

### 3.5. Impact Toughness and the Microstructure Effect

**Figure 11:**
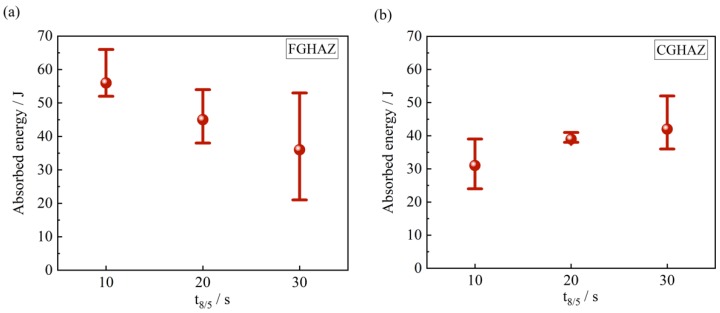
Absorbed energy of simulated (**a**) FGHAZ and (**b**) CGHAZ at the variation of t_8/5_.

The impact toughness of simulated CGHAZ and FGHAZ was present in Figure 11. At −60 °C, the mean absorbed energy of simulated CGHAZ was 42, 39 and 31 J with t_8/5_ of 10 s, 20 s and 30 s, while it was 36, 45 and 56 J in the FGHAZ at the identical cooling rate. The impact toughness seemed to be independent of the cooling time t_8/5_ at the different peak temperatures. In addition, the FGHAZ did not show more superior impact toughness than CGHAZ, which differentiates from conventional high strength low alloy (HSLA) steel. Even much finer effective grain and more 10~11% retained austenite, the impact toughness was not much better in FGHAZ than in CGHAZ. The harder martensite was the main factor in impact toughness. Moreover, the retained austenite may transform into martensite at −60 °C during the impact test, which is referred to as the transformation-induced plasticity (TRIP) effect [19]. This is due to the relatively lower enrichment factor of 1.8 in retained austenite, which means lower stability of retained austenite. The martensite due to the TRIP effect led to the decrease in impact toughness.

## 4. Conclusions

(1) The pre-existing retained austenite in the investigated 5Mn steel enhanced the reversed austenite nucleation number during heating of the welding thermal cycle and led to the relatively small grain size in simulated CGHAZ and FGHAZ.
(2) Austenite growth dominantly took place in the heating process and there was almost no austenite growth in the cooling process above peak temperature during the simulated welding thermal cycle. Therefore, the relatively small size was found in CGHAZ.
(3) The higher transformation rate was measured to be a maximum of 0.98 in the simulated FGHAZ and a maximum of 0.60 in the simulated CGHAZ. Thus, a 10~11% austenite was retained in FGHAZ.
(4) Compared to CGHAZ with the larger grain size, the impact toughness in fine grain FGHAZ was not significantly improved. This was due to the transformation of relatively harder martensite and the TRIP effect during the impact test in simulated FGHAZ. Therefore, how to retain austenite will be the future work of how to improve impact toughness of HAZ.

## Figures and Tables

**Table 1:** Chemical composition of investigated 5Mn steel (wt.%).

C	Si	Mn	S	P	Ni	Cr + Cu + Mo	Al_t_
0.04~0.06	0.2~0.25	4.9~5.2	≤0.0012	≤0.009	0.27~0.30	0.2~0.5	≤0.023

**Table 2:** Mechanical properties of investigated 5Mn steel.

R_el_/MPa	R_m_/MPa	A/%	KV_2_ (−60 °C)/J
645~650	770~775	25~27	200~205

**Table 3:** Continuous cooling transformation temperature at different t_8/5_.

t_8/5_/s	5	7.5	20	30	60	Peak Temperature
Start transformation temperature/°C	371	380	383	388	394	T_p_ = 1320 °C
	397	403	414	405	423	T_p_ = 850 °C
Finish transformation temperature/°C	220	235	212	213	200	T_p_ = 1320 °C
	251	214	256	261	258	T_p_ = 850 °C
Transformation temperature range/°C	151	145	171	175	194	T_p_ = 1320 °C
	146	189	158	144	165	T_p_ = 850 °C
